# Synergistic anticancer activity of combined ATR and ribonucleotide reductase inhibition in Ewing's sarcoma cells

**DOI:** 10.1007/s00432-023-04804-0

**Published:** 2023-04-25

**Authors:** Max-Johann Sturm, Julián Andrés Henao-Restrepo, Sabine Becker, Hans Proquitté, James F. Beck, Jürgen Sonnemann

**Affiliations:** 1 Department of Paediatric and Adolescent Medicine, Jena University Hospital, Friedrich Schiller University Jena, Am Klinikum 1, 07747 Jena, Germany; 2 Research Centre Lobeda, Jena University Hospital, Friedrich Schiller University Jena, Jena, Germany; 3 Placenta Laboratory, Department of Obstetrics, Jena University Hospital, Friedrich Schiller University Jena, Jena, Germany

**Keywords:** Ewing’s sarcoma, ATR, Ribonucleotide reductase, VE821, Triapine, Didox

## Abstract

**Purpose:**

Ewing’s sarcoma is a highly malignant childhood tumour whose outcome has hardly changed over the past two decades despite numerous attempts at chemotherapy intensification. It is therefore essential to identify new treatment options. The present study was conducted to explore the effectiveness of combined inhibition of two promising targets, ATR and ribonucleotide reductase (RNR), in Ewing’s sarcoma cells.

**Methods:**

Effects of the ATR inhibitor VE821 in combination with the RNR inhibitors triapine and didox were assessed in three Ewing’s sarcoma cell lines with different *TP53* status (WE-68, SK-ES-1, A673) by flow cytometric analysis of cell death, mitochondrial depolarisation and cell cycle distribution as well as by caspase 3/7 activity determination, by immunoblotting and by real-time RT-PCR. Interactions between inhibitors were evaluated by combination index analysis.

**Results:**

Single ATR or RNR inhibitor treatment produced small to moderate effects, while their combined treatment produced strong synergistic ones. ATR and RNR inhibitors elicited synergistic cell death and cooperated in inducing mitochondrial depolarisation, caspase 3/7 activity and DNA fragmentation, evidencing an apoptotic form of cell death. All effects were independent of functional p53. In addition, VE821 in combination with triapine increased p53 level and induced p53 target gene expression (*CDKN1A*, *BBC3*) in p53 wild-type Ewing’s sarcoma cells.

**Conclusion:**

Our study reveals that combined targeting of ATR and RNR was effective against Ewing’s sarcoma in vitro and thus rationalises an in vivo exploration into the potential of combining ATR and RNR inhibitors as a new strategy for the treatment of this challenging disease.

**Supplementary Information:**

The online version contains supplementary material available at 10.1007/s00432-023-04804-0.

## Introduction

Ewing's sarcoma (ES) is one of the most aggressive malignancies in children and adolescents (Grünewald et al. 2018; Riggi et al. 2021); less than 10% of ES patients survived before the introduction of chemotherapy. The establishment of intensive treatment consisting of multimodal chemotherapy along with surgery and/or irradiation considerably improved the prognosis, and the 5-years survival for ES is now ~ 70% in high-income countries (Botta et al. 2022). However, the current chemotherapy protocols appear to be optimised to the limits (Koch et al. 2022), and no more improvement in survival was gained over the last two decades (Botta et al. 2022). It is therefore imperative to develop new treatment strategies that promise greater effectiveness and higher cure rates.

In an ongoing effort to systematically assess novel antineoplastic drugs, in particular novel drug combinations, for their potential as ES therapeutics, we found inhibitors of class I/II histone deacetylases (Sonnemann et al. 2007) as well as modulators of sirtuins (Sonnemann et al. 2016; Marx et al. 2018), activators of wild-type p53 (Sonnemann et al. 2011) and inhibitors of polo-like kinase 4 (Kerschner-Morales et al. 2020) to be effective against ES in vitro. We have recently extended this effort to ATR inhibitors (ATRi), aiming at exploiting the replication stress response as a potential treatment regimen for ES (Marx et al. 2021).

ES is driven by a pathognomonic chromosomal rearrangement that fuses an *FET* family gene with an *ETS* family gene, most commonly (i.e., in > 85% of cases) leading to the *EWSR1-FLI1* gene fusion that encodes the EWS-FLI1 oncoprotein (Grünewald et al. 2018; Riggi et al. 2021). EWS-FLI1 is responsible for cancer initiation and maintenance by acting primarily as a neomorphic transcription factor (Perry et al. 2019). It promotes global transcription, thereby elevating R-loops and, in turn, increasing replication stress (Gorthi et al. 2018). The targeting of the latter by inhibiting kinases involved in the DNA damage response, such as ATR, CHEK1 and WEE1, is currently emerging as a promising anticancer approach (Ngoi et al. 2021; da Costa et al. 2023). Since ES tumours frequently exhibit high levels of endogenous replication stress (Nieto-Soler et al. 2016; Goss et al. 2017; Gorthi et al. 2018; Koppenhafer et al. 2020; Soni et al. 2023), they are supposed to be exquisitely vulnerable to replication stress-targeting agents (Martin et al. 2021; Keller et al. 2022).

ATR kinase is a key mediator of the response to replication stress (Saxena and Zou 2022; da Costa et al. 2023; Cybulla and Vindigni 2023), and as such, has attracted much interest as therapeutically exploitable target (Lecona and Fernandez-Capetillo 2018; Bradbury et al. 2020; Cleary et al. 2020). As to ES, ATRi were shown to be effective as single drugs (Nieto-Soler et al. 2016) and to be synergistic with the WEE1 inhibitor adavosertib or the HSP90 inhibitor AUY922 (Koppenhafer et al. 2020; Marx et al. 2021). The ribonucleotide reductase (RNR) is another viable target for the treatment of cancer, as indicated by results from a number of clinical trials (Mannargudi and Deb 2017). The inhibition of RNR, the rate-limiting enzyme in the de novo synthesis of deoxyribonucleotides (Aye et al. 2015; Greene et al. 2020), reduces intracellular nucleotide pools, in this way promoting replication stress (da Costa et al. 2023). RNR has recently turned out to be a promising candidate target for the management of ES, too (Goss and Gordon 2016; Ohmura et al. 2021).

All told, both ATRi and RNR inhibitors (RNRi) hold promise as anti-ES agents. However, single-agent therapy is generally insufficient and, hence, combination therapy is required to achieve durable disease control in nearly all ES cases (Bailey et al. 2019; Zöllner et al. 2021). Replication stress-targeted therapy is very unlikely to make an exception of this rule either (Baxter et al. 2022). We therefore set out to assess if the antineoplastic action of the combination of ATRi with RNRi would be superior to that of either drug alone. We found that ATRi synergised with RNRi in eliciting cell death in ES cells, thus substantiating the therapeutic potential of this drug combination for ES treatment.

## Material and methods

### Cell culture

WE-68 (RRID: CVCL_9717) cells were kindly provided by Dr. F. van Valen (Münster, Germany), SK-ES-1 (RRID: CVCL_0627) cells were purchased from the DSMZ (Braunschweig, Germany), and A673 (RRID: CVCL_0080) cells were purchased from Sigma Aldrich (Deisenhofen, Germany). WE-68 and SK-ES-1 cells were cultured in RPMI 1640 medium, and A673 cells were cultured in DMEM (Capricorn Scientific, Ebsdorfergrund, Germany). Media were supplemented with 10% foetal calf serum (Capricorn Scientific), 100 units/ml penicillin G sodium and 100 µg/ml streptomycin sulphate (Lonza, Basel, Switzerland). Cells were cultured throughout in rat-tail collagen-coated (5 µg/cm^2^; Merck, Darmstadt, Germany) cell culture vessels. Cells were maintained in an incubator at 37 °C and 5% CO_2_ and regularly passaged at a confluence of $\sim$ 90%. Mycoplasma contamination was tested with the qPCR Mycoplasma Test Kit from Applichem (Darmstadt, Germany).

### Treatment of cells

WE-68 and SK-ES-1 cells were cultured in 12-well tissue culture plates, and A673 cells were cultured in 6-well tissue culture plates. For flow-cytometric analyses, cells were seeded at a density of 150,000 cells/well, and for caspase 3/7 assay and PCR analyses, cells were seeded at a density of 200,000 cells/well. For immunoblotting, cells were seeded in 25 cm^3^ tissue culture flasks at a density of 1.5 × 10^6^ cells/flask. Cells were treated with the ATRi VE821 (1–5 µM; Medchem Express, Monmouth Junction, NJ, USA) 1 h before treatment with the RNRi triapine (0.125–1 µM; Medchem Express) or didox (25–100 µM; Cayman Chemical, Ann Arbor, MI, USA) for either 48 h (flow-cytometric analyses) or 24 h (caspase 3/7 activity, immunoblotting, PCR). In the respective experiments, cells were pretreated with the broad-spectrum caspase inhibitor z-VAD-fmk (20 µM; Enzo Life Sciences, Lörrach, Germany) 1 h before treatment with VE821.

### Flow-cytometric analysis of cell death and mitochondrial transmembrane potential (Δ*ψ*_m_) dissipation

Cell death was analysed by assessing the integrity of the cell membrane by flow-cytometric analysis of propidium iodide (PI; Sigma Aldrich) uptake. After harvesting, cells were incubated in 2 µg/ml PI in PBS for 5 min at 4 °C in the dark. The loss of Δ*ψ*_m_ was analysed by determining 3,3′-dihexyloxacarbocyanine iodide (DiOC_6_(3); Thermo Fisher Scientific, Dreieich, Germany) staining of mitochondria. Before harvesting, cells were incubated with 50 nM DiOC_6_(3) for 45 min at 37 °C in the dark. In both read-outs, 10,000 cells per sample were analysed on a BD FACS Canto II (Heidelberg, Germany) using BD FACSDiva software; events were gated to exclude debris and doublets. The results of the cell death determinations were analysed for synergism/antagonism by the combination index (CI) method according to Chou and Talalay using Calcusyn software from Biosoft (Cambridge, UK); CI values of < 1, = 1 and > 1 indicate synergism, additivism and antagonism, respectively (Chou 2006).

### Cell cycle analysis

For cell cycle analysis, ethanol-fixed cells were flow-cytometrically assessed for PI incorporation into DNA. After harvesting, cells were washed twice with PBS and fixed in 70% ethanol at − 20 °C over night. After washing, cells were resuspended in PBS containing 1% glucose, 2.5 µl/ml RNase (Roche, Mannheim, Germany) and 50 µg/ml PI. After 45 min of incubation in the dark, 20,000 cells/sample were analysed on a BD FACS Canto II using BD FACSDiva software; events were gated to exclude debris and doublets.

### Caspase 3/7 activity

Caspase 3/7 activity was determined by measuring the fluorescence of the caspase 3/7 substrate Ac-DEVD-AMC (Bachem, Weil am Rhein, Germany). After harvesting, cells were lysed in 10 mM Tris–HCl, 10 mM NaH_2_PO_4_/NaHPO_4_ (pH 7.5), 130 mM NaCl, 1% Triton X-100 and 10 mM Na_4_P_2_O_7_ for 15 min at 4 °C in the dark. Samples were mixed with 20 mM Hepes (pH 7.5), 10% glycerol, 2 mM DTT and 25 µg/ml Ac-DEVD-AMC. The release of AMC was measured at an excitation of 355 nm and an emission of 460 nm on a Tecan Infinite M200 Pro (Crailsheim, Germany) plate reader. Relative caspase 3/7 activities were calculated as the ratio of the emission of treated to untreated cells.

### Immunoblotting

Cells were centrifuged at 250×g for 5 min and lysed in 200 µl RIPA buffer (Abcam, Cambridge, UK) supplemented with 20 µl/ml protease and phosphatase (Serva Electrophoresis, Heidelberg, Germany) inhibitor cocktails. Sample volumes equivalent to 50 μg of protein were prepared in Laemmli SDS sample buffer (Thermo Fisher Scientific) and incubated at 85 °C for 2 min. 20 µl sample per lane were separated by standard SDS-PAGE on 4–12% precast gels (Serva) and electrophoretically transferred to PVDF membranes (Thermo Scientific). After 1 h blocking in TBS (pH 7.6) containing 5% BSA and 0.1% Tween-20, the membranes were incubated overnight at 4 °C with mouse anti-p53 (Santa Cruz Biotechnology, Heidelberg, #sc-126, RRID: AB_628082; 1:100) antibody. Equal loading of protein was verified by using rabbit anti-GAPDH (Cell Signaling, Danvers, MA, USA, #2118, RRID: AB_561053; 1:3000). HRP-conjugated anti-mouse IgG (Cell Signaling, #7076, RRID: AB_330924; 1:3000) and HRP-conjugated anti-rabbit IgG (Cell Signaling, #7074, RRID: AB_2099233; 1:20,000) were used as secondary antibodies followed by detection of specific signals using Immobilon Forte Western HRP Substrate (Sigma Aldrich). Imaging was done on an MF ChemiBis 3.2 imaging system (DNR Bio Imaging Systems, Neve Yamin, Israel).

### Real-time RT-PCR

All procedures were conducted as per the manufacturers’ instructions. In brief, total RNA was isolated using the Peqgold Total RNA Kit including DNase digestion (Peqlab, Erlangen, Germany). RNA was transcribed into cDNA using the Omniscript RT Kit (Qiagen, Hilden, Germany). Real-time PCR was carried out on a Thermo Fisher Scientific Applied Biosystems 7900HT Real-Time PCR system. Reactions were done in duplicate using Applied Biosystems Gene Expression Assays and Universal PCR Master Mix. *CDKN1A* (#Hs00355782_m1) and *BBC3* (#Hs00248075_m1) expression levels were normalized to *B2M* (#Hs00187842_m1) expression levels, and the relative gene expressions were calculated by the 2(^–ΔΔCt^) method.

### Statistical analysis

Statistical significance of differences between experimental groups was evaluated by the paired two-tailed Student’s *t* test using Microsoft Excel 2016 (*p < 0.05, **p < 0.01, ***p < 0.001).

## Results

### ATRi and RNRi synergise to induce cell death in ES cells

This study aimed at exploring the potential anticancer cooperativity between the ATRi VE821 and the RNRi triapine (3-aminopyridine-2-carboxaldehyde thiosemicarbazone (3-AP)) in cultured ES cells. VE821 was developed as a selective ATRi (Reaper et al. 2011), and its clinical formulation berzosertib (also known as VE822, VX970 and M6620) is being tested in several phase I and II clinical trials (Groelly et al. 2023). Triapine inhibits the M2 subunit of RNR (RRM2; also termed β2) (Greene et al. 2020). It was evaluated in a number of phase I and II studies (Mannargudi and Deb 2017) and is currently being examined in a phase III study of advanced-stage cervical and vaginal cancers (ClinicalTrials.gov Identifier: NCT02466971). We used three ES cell lines with different *TP53* status to test VE821-triapine combination treatment: p53 wild-type WE-68 cells, p53 missense mutant (C176F) SK-ES-1 cells (Sonnemann et al. 2015) and p53 null A673 cells (Ottaviano et al. 2010), to consider a potential impact of p53 on the response of ES cells to the treatment.

**Fig. 1:**
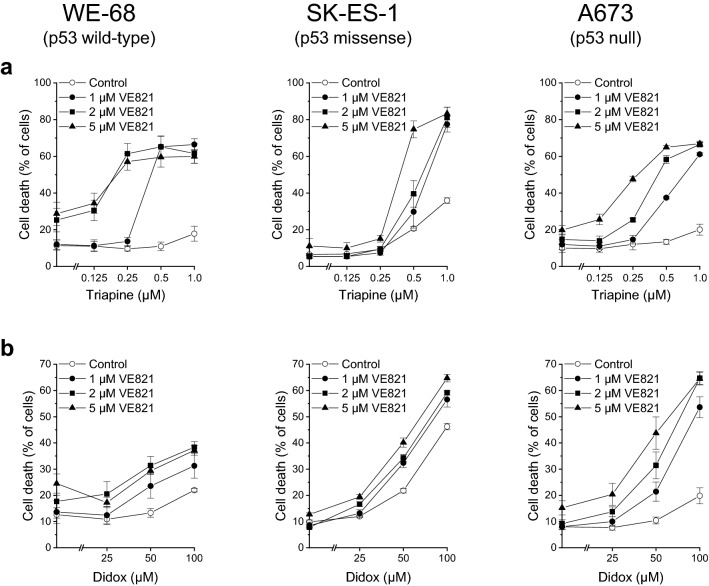
ATRi and RNRi cooperate in inducing cell death in ES cells. One hour after administration of the ATRi VE821, cells were exposed to the RNRi triapine (**a**) or didox (**b**) for another 48 h. Cell death was determined by flow-cytometric analysis of PI uptake. Means ± SEM of each three independent measurements are shown

We initially monitored cell death to examine a possible favourable cytotoxic interaction between VE821 and triapine. One hour after treatment with VE821, cells were exposed to triapine for another 48 h. As shown in Fig. 1a, WE-68 and A673 cells were marginally sensitive to triapine alone under these conditions. Yet, when cells were cotreated with VE821, triapine evoked cell death in a concentration-dependent manner. For example, in combination with VE821 at a concentration of 1 µM—which by itself did not cause any cell death in the three cell lines (see Fig. 1a and b)—triapine-induced cell killing reached 66.5% in WE-68 cells and 61.1% in A673 cells. SK-ES-1 cells were moderately responsive to triapine, viz., treatment with triapine alone resulted in up to 35.9% cell death. In conjunction with VE821, however, triapine-triggered cell death amounted up to 83.3%. We tested the combination of VE821 with triapine for synergy by the CI method (Chou 2006). In WE-68 cells, synergy was seen after all treatment combinations except for the combinations of 1 µM VE821 with 0.125 µM or 0.25 µM triapine (Table 1). In SK-ES-1 cells, all combinations with the two higher concentrations (0.5 µM and 1 µM) of triapine produced synergistic effects (Table 2). In A673 cells, a synergistic interaction was observed in all cases except for 1 µM VE821 combined with 0.125 µM or 0.25 µM triapine, and for 2 µM VE821 combined with 0.125 µM triapine (Table 3).

**Table 1:** CI values for VE821 plus triapine in WE-68 cells

VE821 (µM)	Triapine (µM)	CI
1	0.125	1.893
1	0.25	1.448
1	0.5	**0.023**
1	1.0	**0.021**
2	0.125	**0.429**
2	0.25	**0.058**
2	0.5	**0.045**
2	1.0	**0.058**
5	0.125	**0.809**
5	0.25	**0.192**
5	0.5	**0.163**
5	1.0	**0.160**

Based on data from Fig. 1a, CI values were calculated with the Chou-Talalay method

CI values in bold indicate a synergistic interaction

**Table 2:** CI values for VE821 plus triapine in SK-ES-1 cells

VE821 (µM)	Triapine (µM)	CI
1	0.125	1.876
1	0.25	1.981
1	0.5	**0.629**
1	1.0	**0.156**
2	0.125	2.623
2	0.25	1.665
2	0.5	**0.412**
2	1.0	**0.124**
5	0.125	1.614
5	0.25	1.126
5	0.5	**0.092**
5	1.0	**0.109**

Based on data from Fig. 1a, CI values were calculated with the Chou-Talalay method

CI values in bold indicate a synergistic interaction

**Table 3:** CI values for VE821 plus triapine in A673 cells

VE821 (µM)	Triapine (µM)	CI
1	0.125	1.886
1	0.25	1.068
1	0.5	**0.060**
1	1.0	**0.008**
2	0.125	1.544
2	0.25	**0.258**
2	0.5	**0.008**
2	1.0	**0.005**
5	0.125	**0.449**
5	0.25	**0.037**
5	0.5	**0.007**
5	1.0	**0.007**

Based on data from Fig. 1a, CI values were calculated with the Chou-Talalay method

CI values in bold indicate a synergistic interaction

To confirm the usefulness of RNRi as combination partners for ATRi, we in addition assessed the combination of VE821 with another RNRi, didox. Didox is an inhibitor of the RRM2 subunit, too, and was evaluated in a few phase I and II trials (Mannargudi and Deb 2017). Our assessments of VE821-didox-induced cell death revealed a cooperative effect also of this ATRi-RNRi combination (Fig. 1b). As observed for triapine (compare Fig. 1a), WE-68 and A673 cells were again only modestly responsive to RNR inhibition alone, while SK-ES-1 cells displayed clear responsiveness to didox. At any rate, however, coexposure to VE821 enhanced didox-provoked cell death. The combinatorial effect of VE821 and didox was especially pronounced in A673 cells: when administered alone, didox killed up 19.8% of cells, but when combined with VE821, didox-induced cell death reached 64.7%. The CI analyses validated the synergistic interaction between VE821 and didox at all concentrations except for a few combinations with 25 µM didox (Tables 4, 5 and 6).

**Table 4:** CI values for VE821 plus didox in WE-68 cells

VE821 (µM)	Didox (µM)	CI
1	25	1.975
1	50	**0.611**
1	100	**0.501**
2	25	**0.923**
2	50	**0.388**
2	100	**0.335**
5	25	3.043
5	50	**0.810**
5	100	**0.525**

Based on data from Fig. 1b, CI values were calculated with the Chou-Talalay method

CI values in bold indicate a synergistic interaction

**Table 5:** CI values for VE821 plus didox in SK-ES-1 cells

VE821 (µM)	Didox (µM)	CI
1	25	1.011
1	50	**0.740**
1	100	**0.695**
2	25	**0.818**
2	50	**0.695**
2	100	**0.642**
5	25	**0.759**
5	50	**0.577**
5	100	**0.537**

Based on data from Fig. 1b, CI values were calculated with the Chou-Talalay method

CI values in bold indicate a synergistic interaction

**Table 6:** CI values for VE821 plus didox in A673 cells

VE821 (µM)	Didox (µM)	CI
1	25	1.152
1	50	**0.476**
1	100	**0.131**
2	25	**0.872**
2	50	**0.254**
2	100	**0.074**
5	25	**0.635**
5	50	**0.142**
5	100	**0.078**

Based on data from Fig. 1b, CI values were calculated with the Chou-Talalay method

CI values in bold indicate a synergistic interaction

### The synergistic activity of ATRi and RNRi involves apoptosis

**Fig. 2:**
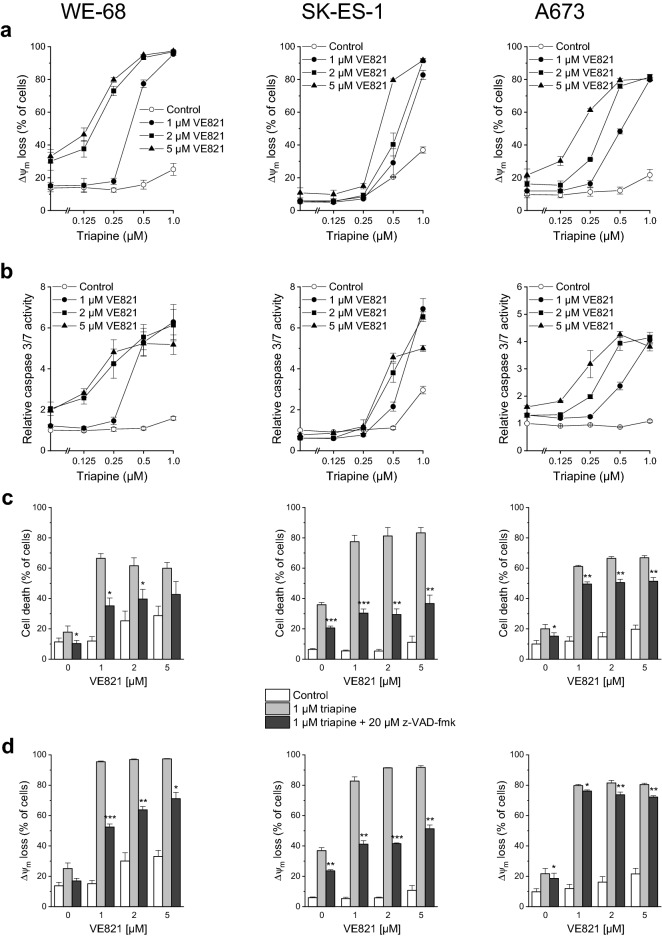

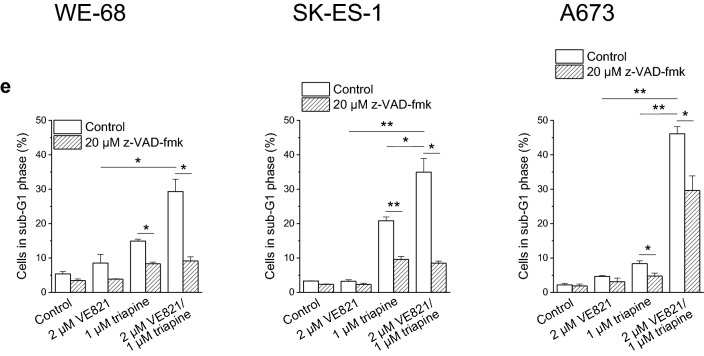
The ATRi VE821 and the RNRi triapine cooperate in inducing apoptosis in ES cells. One hour after administration of VE821, cells were exposed to triapine for another 24 h (**b**) or 48 h (**a, c, d, e**). **c, d, e** z-VAD-fmk was applied 1 h before treatment with VE821. **a, d** Loss of Δ*ψ*_m_ was determined by flow-cytometric analysis of DiOC_6_(3) staining. **b** Caspase 3/7 activity was determined using the fluorogenic substrate Ac-DEVD-AMC; relative caspase 3/7 activities are the ratio of treated cells to untreated cells. **c** Cell death was determined by flow-cytometric analysis of PI uptake. **e** sub-G1 cells were determined by flow-cytometric cell cycle analysis of PI-stained ethanol-fixed cells. Means ± SEM of each three independent measurements are shown (*p < 0.05, **p < 0.01, ***p < 0.001)

Although cell death can proceed via various pathways, anticancer drugs typically engage the apoptotic one (Bhola and Letai 2016). Thus, we asked whether the synergistic anticancer action of ATRi-RNRi combination treatment also involved apoptosis. To this end, we examined Δ*ψ*_m_ decay, caspase 3/7 activity as well as the effect of the broad-spectrum caspase inhibitor z-VAD-fmk on VE821-triapine-elicited cell death, Δ*ψ*_m_ decay and DNA fragmentation. In accord with our cell death findings, triapine alone had a modest effect on Δ*ψ*_m_ in WE-68 and A673 cells (Fig. 2a). When the same experiment was carried out in cells coexposed to VE821, however, triapine evoked Δ*ψ*_m_ dissipation in up to 97.3% of WE-68 cells and in up to 81.5% of A673 cells. Noncotreated SK-ES-1 cells showed some responsiveness to triapine (i.e., 36.9% loss of Δ*ψ*_m_ after 1 µM triapine), whereas VE821-cotreated SK-ES-1 cells displayed a very strong one (i.e., up to 91.7% loss of Δ*ψ*_m_ after 1 µM triapine). Very similar effect patterns were found in the caspase 3/7 activity measurements: VE821 or triapine applied individually activated caspase 3/7 only weakly, and their combination produced potentiated effects (Fig. 2b). In line with these results, z-VAD-fmk significantly reduced VE821-triapine-triggered cell death in the three cell lines, albeit to different extents: the pan-caspase inhibitor had a substantial effect in WE-68 and SK-ES-1 cells, yet an only weak one in A673 cells (Fig. 2c). Likewise, z-VAD-fmk markedly alleviated VE821-triapine-mediated Δ*ψ*_m_ dissipation in WE-68 and SK-ES-1 cells, but did much less so in A673 cells (Fig. 2d).

To further substantiate that the combination of ATRi with RNRi elicited apoptosis, we determined the sub-G1 fraction of cells with fragmented DNA < 2*n* by cell cycle analysis. As illustrated in Fig. 2e, VE821-triapine induced DNA fragmentation in the three cell lines. Yet again, z-VAD-fmk demonstrated different effectiveness: it prevented DNA fragmentation in WE-68 and SK-ES-1 cells, while it was only moderately effective in A673 cells. The cell cycle analyses also served to assess the impact of VE821 and triapine on cell cycle phase distribution. VE821 alone unsurprisingly caused an accumulation of cells in the G1 phase, while triapine alone increased the G2/M phase fraction of cells (Fig. S1). Remarkably, VE821-triapine combination treatment blocked cell cycle progression to the G2/M phase. It should be noted, however, that triapine treatment resulted in cell cycle profiles without a clear separation of G1, S and G2/M populations (Fig. S2), rendering the quantification of cell cycle phases challenging in these cases.

**Fig. 3:**
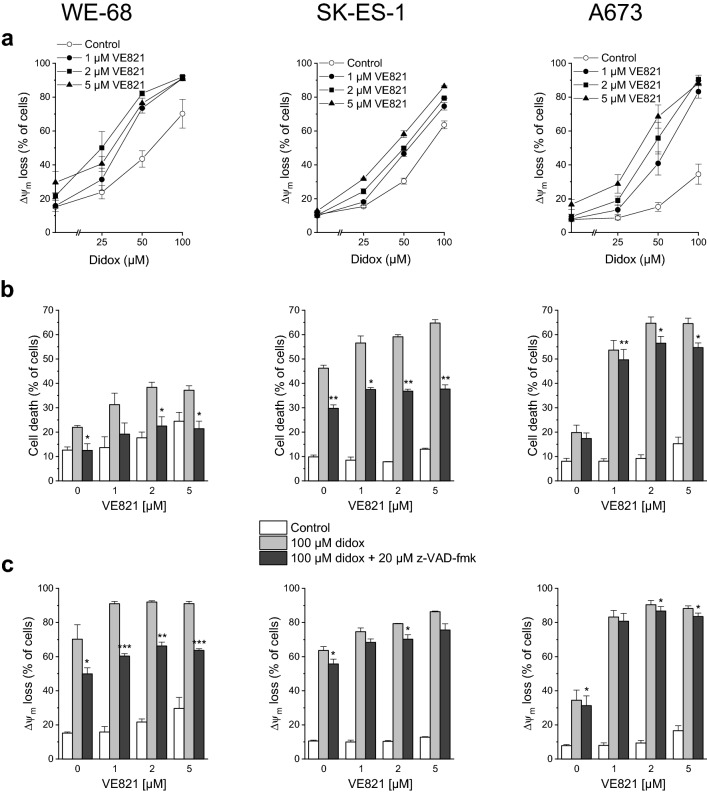
The ATRi VE821 and the RNRi didox cooperate in inducing apoptosis in ES cells. One hour after administration of VE821, cells were exposed to didox for another 48 h. **b, c** z-VAD-fmk was applied 1 h before treatment with VE821. **a, c** Loss of Δ*ψ*_m_ was determined by flow-cytometric analysis of DiOC_6_(3) staining. **b** Cell death was determined by flow-cytometric analysis of PI uptake. Means ± SEM of each three independent measurements are shown (*p < 0.05, **p < 0.01, ***p < 0.001)

We likewise tested the combination of VE821 with didox for the induction of apoptosis. As demonstrated in Fig. 3a, didox triggered Δ*ψ*_m_ decay, and VE821 amplified this effect. Like in the analyses of VE821-didox-elicited cell death (compare Fig. 1b), strongest amplification was seen in A673 cells. z-VAD-fmk had a considerable impact on VE821-didox-mediated cell death in WE-68 and SK-ES-1 cells, but a weak one in A673 cells (Fig. 3b), in keeping with its differential impact on VE821-triapine-evoked cell death (compare Fig. 2c). With regard to VE821-didox-induced Δ*ψ*_m_ dissipation, a substantial effect of z-VAD-fmk was observed only in WE-68 cells (Fig. 3c).

### VE821-triapine combination treatment activates p53

**Fig. 4:**
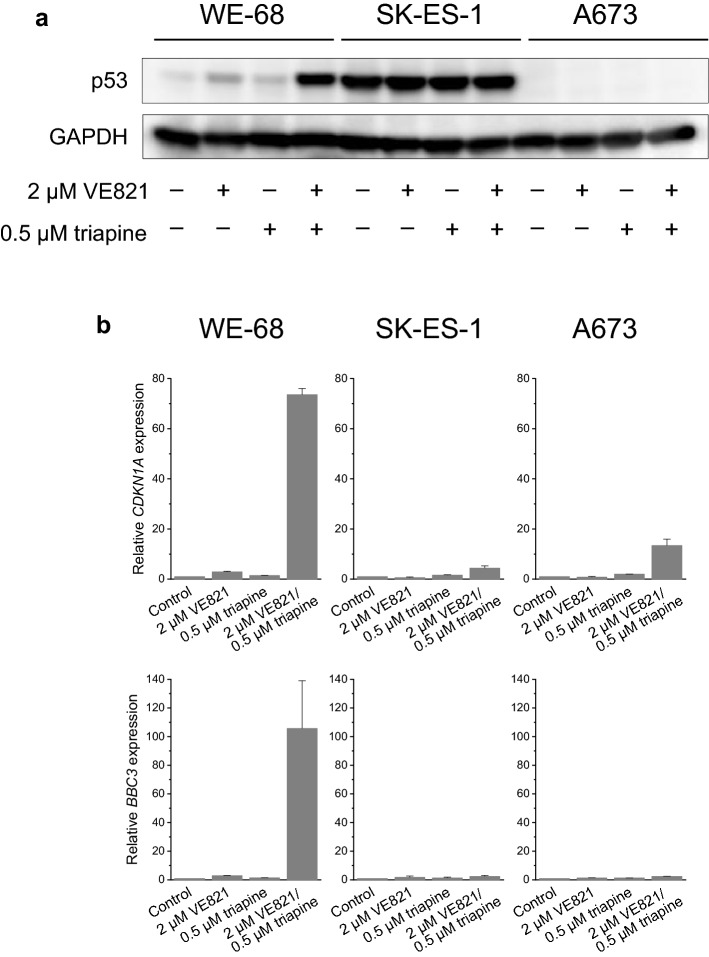
The ATRi VE821 and the RNRi triapine cooperate in increasing p53 level in p53 wild-type ES cells and in inducing p53 target gene expression in ES cells. One hour after administration of VE821, cells were exposed to triapine for another 24 h. **a** The abundance of p53 was determined by immunoblotting; the figure is representative of three independent determinations. **b** *CDKN1A* and *BBC3* expression levels were determined by real-time RT-PCR and normalised to *B2M* expression levels; relative expression levels are the ratio of treated cells to untreated cells. Means ± SEM of each two independent measurements are shown

About 90% of ES tumours harbour wild-type *TP53* (Thoenen et al. 2019). Hence, and in view of the key role of p53 in human cancers (Levine 2020), we evaluated if VE821 and triapine had an effect on p53 in p53 wild-type ES cells. Immunoblot detection revealed a moderate rise of p53 abundance after exposure to VE821 alone and a very strong one after exposure to VE821-triapine in WE-68 cells (Fig. 4a). Consistent with the high expression of mutant p53 found in a wide array of cancers (Bartek et al. 1991), p53 missense mutant SK-ES-1 cells exhibited a pronounced constitutive expression of p53 that, however, was not further increased by the treatment. To corroborate the p53-activating effect of VE821-triapine in WE-68 cells, we determined the mRNA expression of two major p53 target genes, *CDKN1A* (coding for the cell cycle-inhibitory protein p21^WAF1/CIP1^) and *BBC3* (coding for the proapoptotic BCL2 family protein PUMA) (Fischer 2017). As shown in Fig. 4b, VE821-triapine boosted *CDKN1A* expression most notably in the p53 wild-type cells (i.e., 73.6-fold), but also to some degree in the p53 mutant ones (i.e., 4.4-fold in SK-ES-1 cells and 13.4-fold in A673 cells). Similarly, *BBC3* expression was massively induced by VE821-triapine in WE-68 cells (i.e., 105.7-fold), yet only slightly in SK-ES-1 and A673 cells (i.e., 2.4- and 2.5-fold, respectively). In agreement with its moderate effect on p53 abundance, VE821 alone produced also a moderate increase in *CDKN1A* and *BBC3* expression (i.e., both 2.9-fold) in WE-68 cells.

## Discussion

The potential usefulness of combined ATR and RNR inhibition has been evaluated in a number of studies on diverse adult cancers (Liu et al. 2017; Fordham et al. 2018; Konstantinopoulos et al. 2020; Middleton et al. 2021). For all we know, our study is the first one to explore the combination of ATRi with RNRi in a childhood cancer. In this study, we found that the ATRi VE821 cooperated with the RNRi triapine and didox to promote anti-ES activities, and CI analysis showed that the cooperation was synergistic. We further found that the combination of ATRi with RNRi was similarly effective in p53 wild-type and p53 mutant ES cells, a welcome finding as *TP53* mutations predict a poor outcome in ES patients (Crompton et al. 2014; Tirode et al. 2014), including increased radioresistance (Casey et al. 2021). The utility of ATR pathway inhibitors combined with RNRi for the treatment of ES is also supported by reports on the cooperative action of inhibitors of the ATR downstream effector kinases CHEK1 and WEE1 with RNRi in ES (Goss et al. 2017; Koppenhafer et al. 2018; Ohmura et al. 2021).

The RRM2 subunit of RNR was recently identified as a promising target for ES by showing that high *RRM2* expression was associated with poor overall survival in ES patients (Ohmura et al. 2021). Ohmura et al. further showed that triapine inhibited ES growth in vitro and in vivo. Yet they also observed that prolonged treatment with triapine led to relative resistance towards the agent (Ohmura et al. 2021), pointing to the probable insufficiency of triapine monotherapy and thus underscoring the need for combination strategies. Our and other studies (Goss et al. 2017; Koppenhafer et al. 2018; Ohmura et al. 2021) suggest that the combination of RNRi with ATR pathway inhibitors may be a viable option for the treatment of ES. The mechanism that underlies the favourable interaction of this inhibitor combination has not yet been definitively resolved but has been hypothesised to involve a feedback loop in ES cells in which the inhibition of the ATR pathway results in the degradation of the RNR subunit RRM2 (Koppenhafer et al. 2020). This hypothesis is backed by the observation that ATR promotes RRM2 stabilisation by downregulation of the RRM2-degrading SCF(cyclin F) ubiquitin ligase complex (D’Angiolella et al. 2012).

Two mechanistic insights emerge from our study. First, it reveals that the mitochondrial pathway of apoptosis was involved in mediating the synergy of combined ATR and RNR inhibition in ES cells, as judged by assessing a number of features characteristic of apoptosis. We found that ATRi in combination with RNRi led to dissipation of Δ*ψ*_m_—indicating that ATRi-RNRi harnessed the mitochondrial pathway of apoptosis—the activation of caspase 3/7 and the accumulation of sub-G1 cells. These apoptosis read-outs displayed a similar pattern, supporting the robustness of the results. Induction of apoptosis by ATRi-RNRi was further confirmed by the use of z-VAD-fmk, which allows to distinguish caspase-dependent from caspase-independent cell death (Chipuk and Green 2005): the pan-caspase inhibitor prevented DNA fragmentation and cell death to some extent, evidencing a partially caspase-dependent cell death mechanism induced by ATRi-RNRi. Remarkably, z-VAD-fmk also impinged on ATRi-RNRi-elicited Δ*ψ*_m_ decay, suggesting that the mitochondrial apoptotic function hinged, to some extent, on caspases, possibly as a consequence of a feedback activation loop (McComb et al. 2019). Yet it should also be noted that z-VAD-fmk protected cells only incompletely, pointing to a caspase-independent portion of cell death.

Second, our study reveals that concomitant ATR and RNR inhibition resulted in a marked cooperative induction of p53 and p53 target genes in p53 wild-type ES cells. ATRi in combination with RNRi thus may function via the p53 pathway in p53 wild-type cells. It should be taken into account, however, that ATRi-RNRi upregulated *CDKN1A* and *BBC3* also in p53 mutant ES cells, albeit to a much lesser extent. Hence, VE821-triapine did not only kill ES cells irrespective of their *TP53* status, but also its effect on gene expression did not depend on p53. Of note, we do not wish to imply here that the synergistic action of VE821-triapine stemmed from inducing *CDKN1A* and/or *BBC3* expression (although it is tempting to speculate that the massive upregulation of the proapoptotic *BBC3* by VE821-triapine in WE-68 cells contributed to the synergistic apoptosis induction by VE821-triapine in these cells). Rather, these genes served as a tool to find out whether ATRi combined with RNRi acted at the level of gene expression.

Both ATRi and RNRi are promising antineoplastic agents. Here, we have shown that their anticancer effectiveness could be greatly enhanced by combining them. In particular, our in vitro findings establish the combination of ATRi with RNRi as an option for the treatment of ES and provide a rationale for an in vivo exploration into the therapeutic potential of this drug combination.

## Supplementary Information

Below is the link to the electronic supplementary material.

Supplementary file1 (PPTX 751 KB)

## Data Availability

All data sets on which the conclusions of the manuscript rely are presented in the paper and its supplementary information files.
