# Supplementary material for: Synergistic anticancer activity of combined ATR and ribonucleotide reductase inhibition in Ewing's sarcoma cells
Source: J Cancer Res Clin Oncol. 2023 Apr 25;149(11):8605–17. doi: 10.1007/s00432-023-04804-0 (PMC10374484; doi:10.1007/s00432-023-04804-0)
Supplement: Supplementary file 1 — Supplementary file1 (PPTX 751 KB) [file 432_2023_4804_MOESM1_ESM.pptx]

## Slide 1
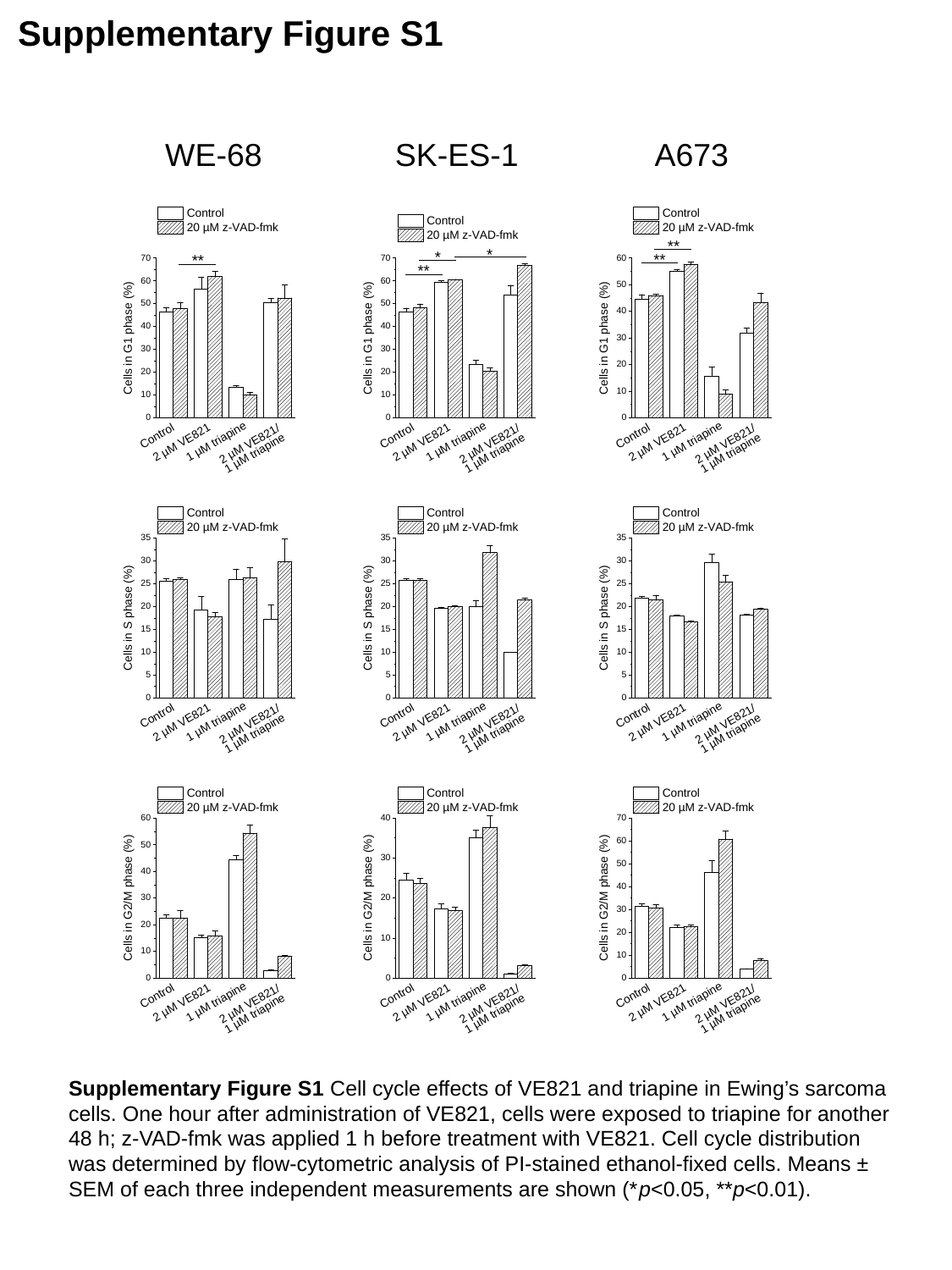

Supplementary Figure S1
WE-68
SK-ES-1
A673
Supplementary Figure S1 Cell cycle effects of VE821 and triapine in Ewing’s sarcoma cells. One hour after administration of VE821, cells were exposed to triapine for another 48 h; z-VAD-fmk was applied 1 h before treatment with VE821. Cell cycle distribution was determined by flow-cytometric analysis of PI-stained ethanol-fixed cells. Means ± SEM of each three independent measurements are shown (*p<0.05, **p<0.01).

## Slide 2
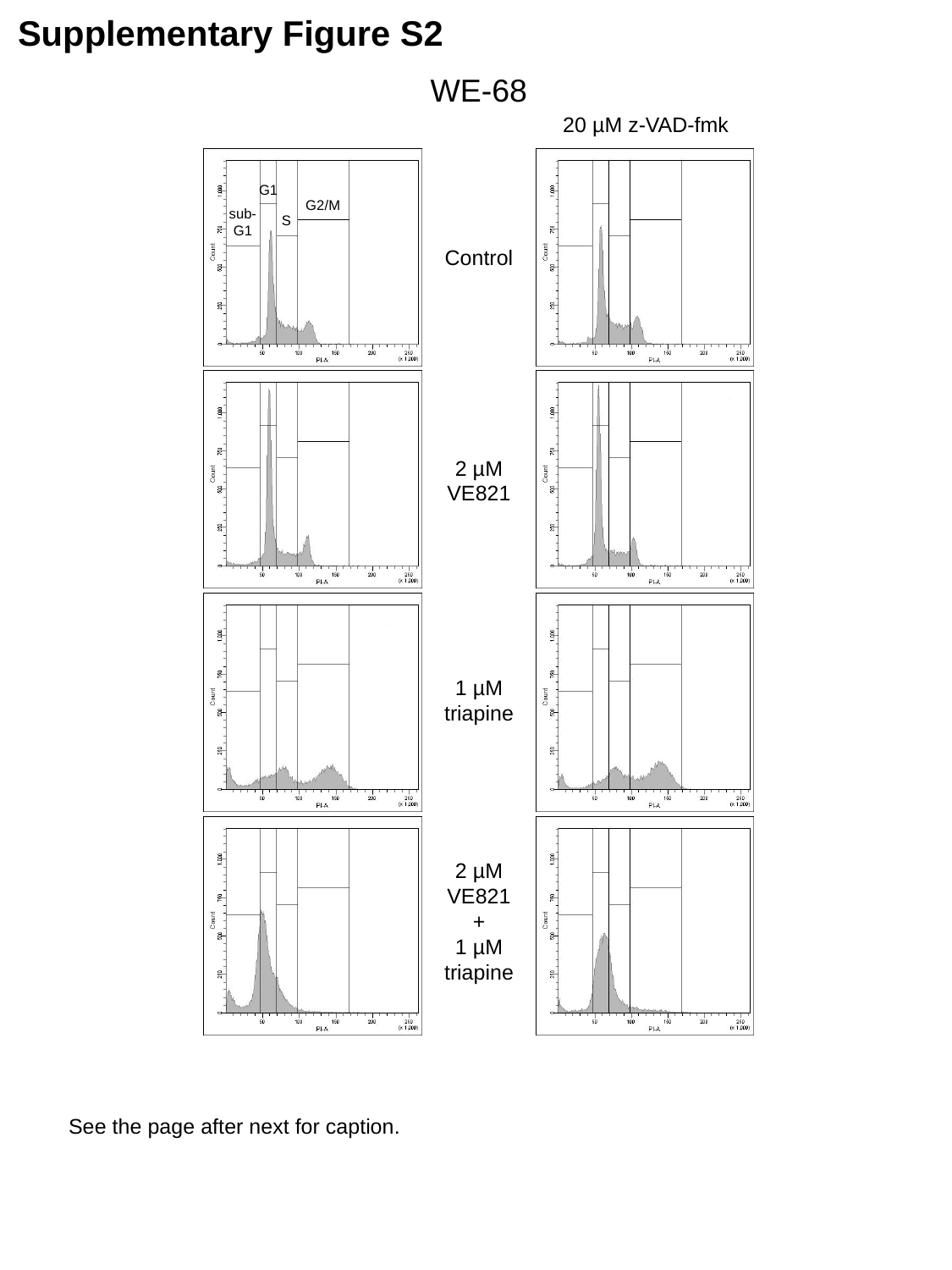

Supplementary Figure S2
WE-68
20 µM z-VAD-fmk
G1
G2/M
sub-
G1
S
Control
2 µM
VE821
1 µM
triapine
2 µM
VE821
+
1 µM
triapine
See the page after next for caption.

## Slide 3
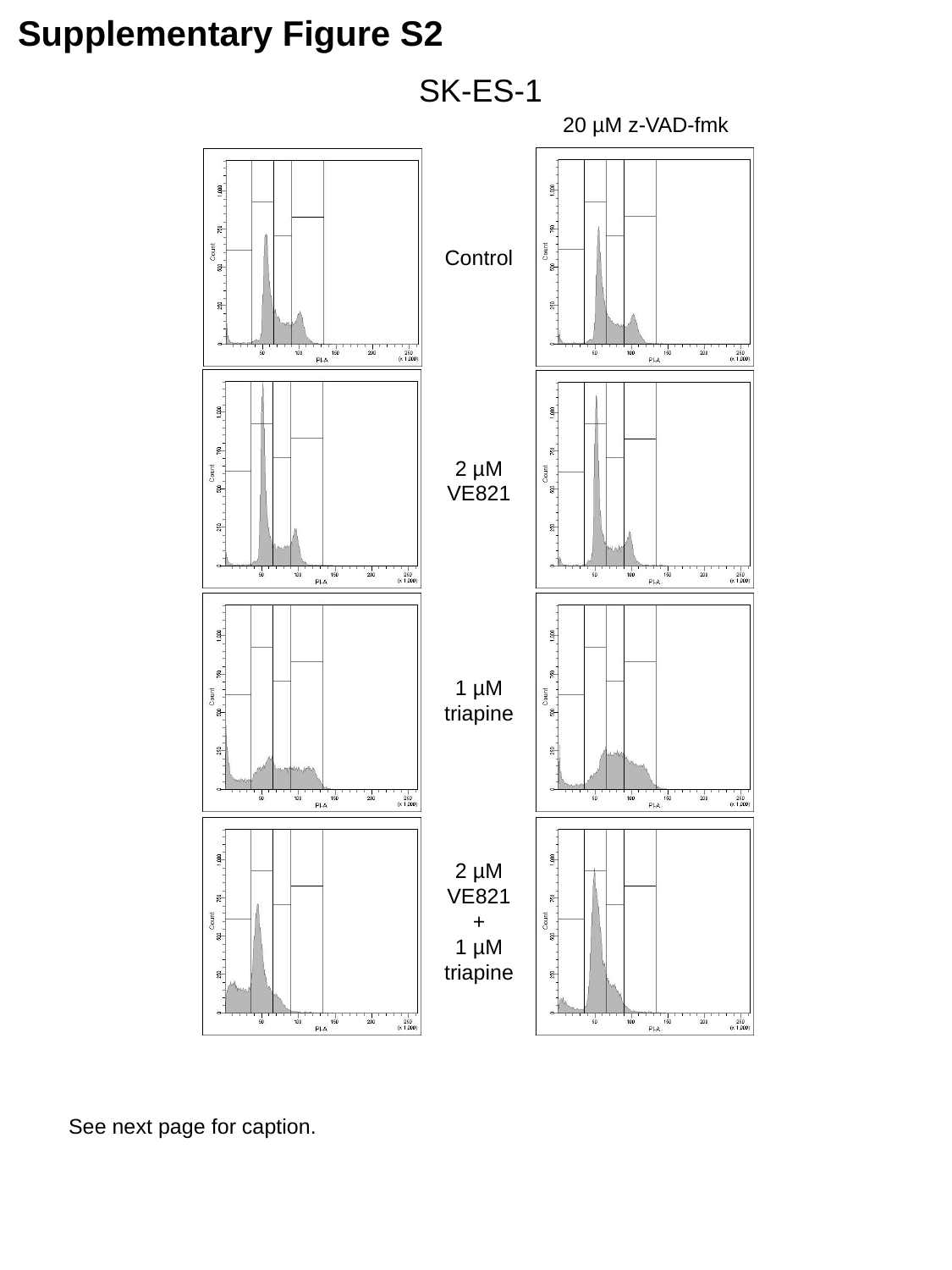

Supplementary Figure S2
SK-ES-1
20 µM z-VAD-fmk
Control
2 µM
VE821
1 µM
triapine
2 µM
VE821
+
1 µM
triapine
See next page for caption.

## Slide 4
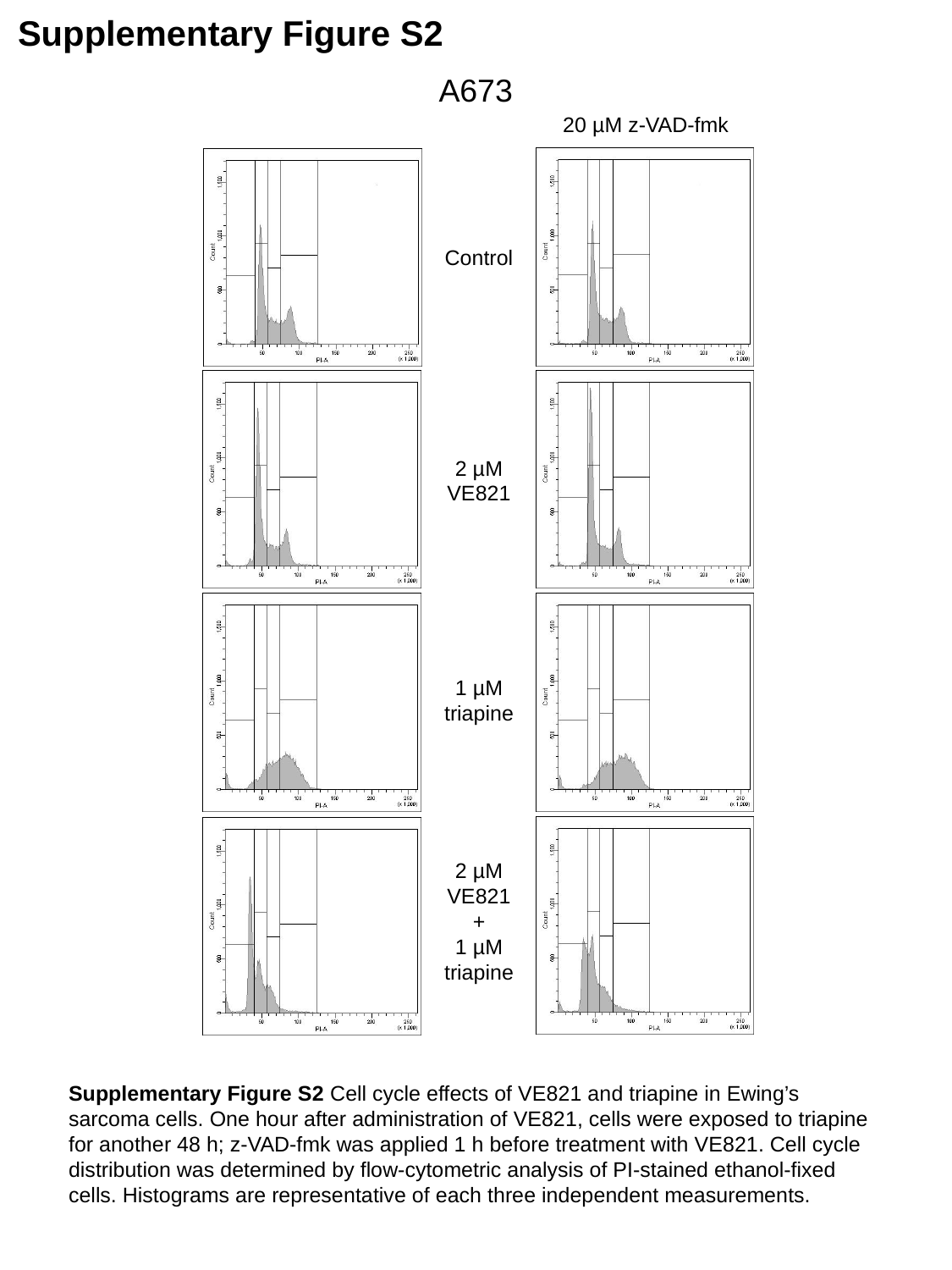

Supplementary Figure S2
A673
20 µM z-VAD-fmk
Control
2 µM
VE821
1 µM
triapine
2 µM
VE821
+
1 µM
triapine
Supplementary Figure S2 Cell cycle effects of VE821 and triapine in Ewing’s sarcoma cells. One hour after administration of VE821, cells were exposed to triapine for another 48 h; z-VAD-fmk was applied 1 h before treatment with VE821. Cell cycle distribution was determined by flow-cytometric analysis of PI-stained ethanol-fixed cells. Histograms are representative of each three independent measurements.
